# Investigation of Notch-Induced Precise Splitting of Different Bar Materials under High-Speed Load

**DOI:** 10.3390/ma13112461

**Published:** 2020-05-28

**Authors:** Yuanzhe Dong, Yujian Ren, Shuqin Fan, Yongfei Wang, Shengdun Zhao

**Affiliations:** 1 School of Mechanical Engineering, Xi’an Jiaotong University, Xi’an 710049, China; renyujian@stu.xjtu.edu.cn (Y.R.); sunnyfan@mail.xjtu.edu.cn (S.F.); wangyongfei324@xjtu.edu.cn (Y.W.); 2 School of Mechanical Engineering, Shaanxi University of Technology, Hanzhong 723001, China

**Keywords:** metal bar, precise cropping, circumferential notch, high-speed

## Abstract

A notch-induced high-speed splitting method was developed for high-quality cropping of metal bars using a new type of electric-pneumatic counter hammer. Theoretical equations and FE models were established to reveal the crack initiation and fracture mode. Comparative tests were conducted for notched and unnotched bars of four types of steels, i.e., AISI 1020, 1045, 52100, and 304, and the section quality and microfracture mechanism were further investigated. The results show that damage initiates at the bilateral notch tips with peak equivalent plastic strain, and propagates through the plane induced by the notch tip; the stress triaxiality varies as a quasi-sine curve, revealing that the material is subjected to pure shearing at the notch tip, and under compression at the adjacent region. High precision chamfered billets were obtained with roundness errors of 1.1–2.8%, bending deflections of 0.5–1.5mm, and angles of inclination of 0.7°–3.4°. Additionally, the notch effectively reduced the maximum impact force by 21.6–23.9%, splitting displacement by 7.6–18.6%, and impact energy by 27.8–39.1%. The crack initiation zone displayed quasi-parabolic shallow dimples due to shear stress, and the pinning effect was larger in AISI 52100 and 1045 steel; the final rupture zone was characterized by less elongated and quasi-equiaxial deeper dimples due to the combination of shear and normal stress.

## 1. Introduction

The precise cropping of blanks from long metal bars is the primary process of near-net-shape forming technologies, such as die-forging and cold-extrusion, and is widely used in the production of most mechanical parts. The industrial cropping process takes place at a loading speed from almost zero to 0.4 m/s. The bar material first experiences plastic deformation under bending, which deviates the maximum shearing stress plane from the vertical cropping direction. After that, cracks initiate and propagate from the stress concentration zones around the edges of the upper and lower blades, producing fracture planes with draw-in distortion, which usually necessitates a further edge-cutting procedure. Therefore, the point of precise cropping is to limit first-stage plastic deformation.

A great deal of effort has been expended on the improvement of section quality. The proposed precise cropping technologies can be classified into six basic types: radially constrained cropping, axially pressured cropping, torsion combined cropping, low-load cyclic splitting, high-speed cropping, and warm cropping at blue-brittleness temperature. In the first two methods, metal bars are circumferentially or axially clamped under large force to avoid bending, thus producing flat and vertical sections, but the roundness error is inferior due to draw-in deformation around the cropped faces [1,2], and the axially pressured cropping method is only feasible for soft metals, such as copper and aluminum [3,4]. Torsion combined cropping [5] significantly reduces bending deflection and improves the roundness of wires with a diameter of 1.96 mm, but leaves ring marks on the fracture plane; additionally, it is still poorly-suited to bars with 10–20mm diameters, because a large torque is required. The low-load cyclic splitting method prefabricates V-shaped notches on the workpiece surface to create a local stress concentration, and exerts a cyclic load to split the workpiece, which changes the irregular ductile fracture into a controlled brittle fracture and yields high-quality flat sections [6,7,8]. The optimal notch depth is suggested to be 4–5% of the bar diameter, with an opening angle of 60–90°, and a tip radius of 0.1–0.2 mm [9]. However, the splitting efficiency is relatively low (3~6 blanks per minute).

The high-speed cropping method enhances the brittleness of metals by increasing the loading speed. For body-centered cubic (BCC) materials, especially mild and medium steels, Armstrong and Walley [10] showed that the yield stress increases with increasing strain rate, while the strain at the maximum tensile stress point decreases, leading to the deformation in a less ductile -mode. Singh et al. [11] compared the dynamic behavior of mild steel at a strain rate of 750 s^-1^ to a quasi-static condition, and found the increase of yield strength was greater (2.5 times) than the ultimate tensile strength (1.18 times). Hor et al. [12] showed that the stress rate sensitivity becomes important at 1000 s^−1^ in cold shear tests on AISI 4140 steel; the self-heating level was measured with an increase of approximately 120 °C at the surface. Lu and Zhu [13,14] pointed out that when the strain rate exceeds 3000 s^−1^, the thermal softening effect shows more influence than strain hardening because of the adiabatic temperature rise. Figiel et al. [15] presented a numerical analysis with finite contact elements from a compressive shear fracture test (CSFT) on layered composite materials, which considered the frictional contribution to critical energy release, and improved the determination of Mode-II fracture toughness and crack propagation analysis. Although the details of the fracture mechanisms remain to be determined, high-speed cropping experiments conducted by Organ [16] on a petrol-forge hammer (7.3–9.5 m/s), and by Chen [17] on an air hammer (4.5 m/s), showed that the cropping quality can be improved for various kinds of steels at room temperature. Song et al. [18] also obtained comparatively flat cropping sections on a 4 kJ hydraulic-pneumatic hammer for low and middle carbon steel bars at a loading speed of 3.5–7 m/s and a temperature of 350–380 °C. However, these high-speed cropping methods have their drawbacks, such as large impact load and energy, and frequent breakage of blades; additionally, there are still obvious distortion zones left on the cropped surface. Also, the aforementioned types of high-energy rate impact equipment are usually very large with a single-direction loading layout which produces a relatively high level of ground vibrations, and presents problems of low energy utilization and complex control of the hydraulic system for the return stroke.

Therefore, a notch-induced, high-speed splitting method was developed which processes V-shape circumferential notches in batches on the bar surface to achieve stress concentration, and applies a high-speed load to complete splitting with a new electric-pneumatic counter hammer system. The theoretical equations of critical load and fracture energy have been obtained, and an FE numerical analysis gives a better understanding of the crack initiation and fracture mode. Comparative tests were conducted for notched and unnotched bars of four types of steel, i.e., low-carbon AISI 1020, medium-carbon 1045, high-carbon 52100 bearing steel, and 304 stainless steel, to investigate the application range of this new method. These steels are widely used in the machinery and automotive industries. Currently, the 1020 and 1045 steel bars with a diameter less than 50 cm are usually cropped by traditional shearing methods, which is highly efficient but causes obvious draw-in distortion. AISI 52100 and 304 steel are typical hard-to-cut steels; the former has high strength [19], and the latter has a high fracture toughness (J_IC_ > 150 kJ/m^2^ ) and good ductility [20]. Both types are usually cropped by sawing or the hot-shearing method. 

## 2. The Method of Notch-induced High-speed Splitting

**Figure 1:**
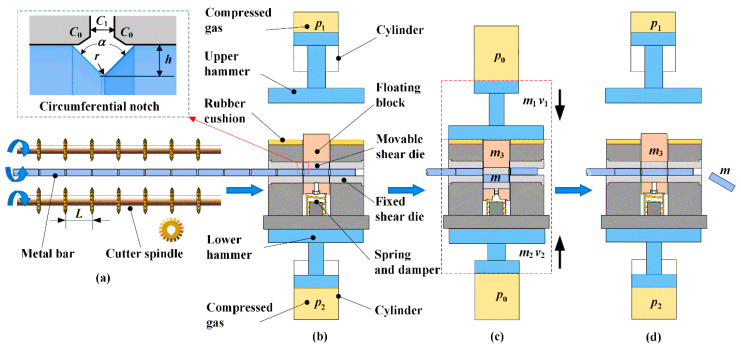
Schematic of the notch-induced, high-speed impact splitting method. (**a**) Batch processing of equally spaced V-shape circumferential notches. (**b**) Energy storage in compressed gas. (**c**) Counter impact stoke of hammers and bar splitting. (**d**) Return stroke of hammers and blank unloading.

The working principle of the new splitting method is illustrated in Figure 1. Firstly, dozens of V-shape circumferential notches are simultaneously processed on the metal bar surface by equally spaced disc cutters according to the splitting length of *L*. As shown in Figure 1a, the two cutter shafts are symmetrically arranged beside the bar, and synchronously rotate clockwise at high speed, while the metal bar rotates anticlockwise at low speed, so that entire circumferential notches are obtained in one semicircle. As shown in Figure 1b, the upper and lower hammers are locked by pneumatic clutches in the initial positions, and the gas inside the cylinders is compressed to store potential energy on both sides, i.e., with pressure *p*_1_ and *p*_2_. The notched bar is radially constrained in the double splitting die, and the first two notches are placed between the movable shear die and the fixed shear die. In the impact stroke, as shown in Figure 1c, the upper hammer strikes downward and impacts the floating block at initial speed *v*_1_, and simultaneously, the lower hammer pushes the whole splitting die upward at speed *v*_2_, so that two blanks are cropped at a time according to the position of the notches. In the return stroke, as shown in Figure 1d, the hammers return to their initial positions, the floating block rebounds by the spring-damper component, and feeding goes on to eject the two cropped blanks.

This new method integrates the technologies of radial constraints to avoid bending and high-speed impact to enhance metal brittleness. The batch processing of the surface notch is proposed before high-speed cropping, as it introduces stress-concentrations at the notch tips to restrain the first-stage plastic deformation of the adjacent material, changing the irregular ductile fracture into a controllable, quasi-brittle fracture. It also reduces the impact force and fracture energy compared to the traditional high-speed shearing method. All the edges of the shear dies are chamfered of *C*_0_ to avoid blade breakage, which is different from the traditional cropping method that needs sharp blades to penetrate and cut off the workpiece. Also, a counter load is exerted, which effectively reduces the level of ground vibration compared to the one-directional, high-speed shearing method.

The geometric profile of the V-shape notch is illustrated with depth *h*, opening angle *α*, and tip radius *r*. The axial clearance of the movable shear die and fixed shear die is *C*_1._ Geometrically, notch width *w* is approximately calculated as w=2htan(α/2) for small tip radius *r*, and needs to satisfy $w\geq C_{1}+2C_{0}$ to avoid burrs being squeezed on the exterior surface. The radial clearance between the bar and the shear dies should be as small as possible to avoid bending; this is practically adjusted to 0.1–0.2 mm, based upon the feasibility of feeding.

**Figure 2:**
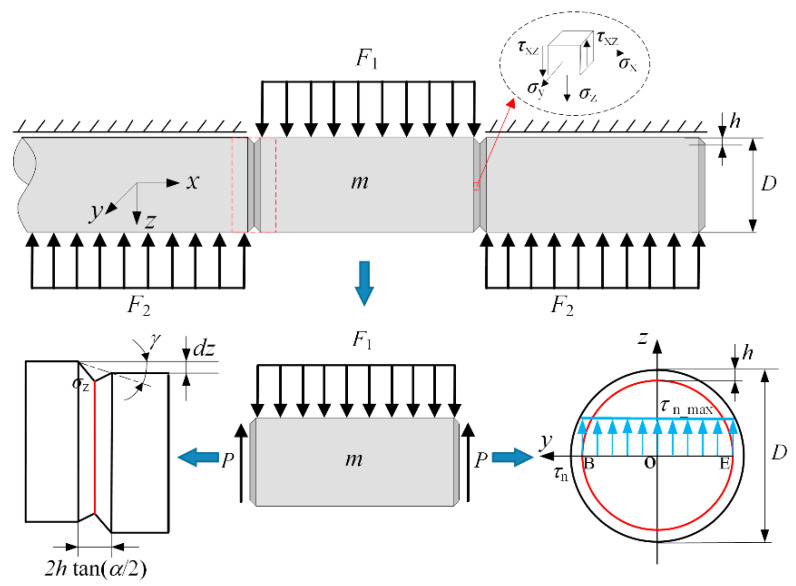
Mechanics diagram of the bar with circumferential notches under symmetrical shear load.

In the splitting stage, as illustrated in Figure 2, time-dependent force *F*_1_ and *F*_2_ drive a counter-motion along the *z*-direction, and a pair of resistant shear force *P* is produced on both sides of the fracture plane. The force equilibrium for the off-cut segment is given, without taking gravity into account [16]

(1) $$F_{1}-2P=m\frac{d^{2}z}{dt^{2}}$$

In the case of a three-directional compression stress state, quasi-pure shearing is liable to occur [18]; thus, shear stress plays a major role in the crack initiation and fracture. Theoretically, the maximum nominal shear stress *τ*_n_ at the neutral plane is expressed as

(2) $$\tau_{n\text{\_}\max}=\frac{4P}{3\pi {\left(D/2-h\right)}^{2}}$$

where *D* is bar diameter and *h* is the notch depth.

Considering the stress concentration, the real maximum shear stress *τ*_max_ at notch tips B and E is defined as follows.

(3) $$\tau_{\max}=k_{t}\frac{4P}{3\pi {\left(D/2-h\right)}^{2}}$$

where *k_t_* is the stress concentration factor. For the round bar with a shallow notch subjected to shear load, *k_t_* is determined as [21,22,23]:

(4) $$k_{t}=(1+\sqrt{\frac{h}{r_{}}})/\phi (\bar{\alpha })$$

where $\bar{\alpha }=\pi -\pi \alpha /180$, and *α* is the opening angle in degree form; *r* is the notch tip radius and $\phi (\bar{\alpha })$ is given based on a finite element analysis [22,23]

(5) $$\phi (\bar{\alpha })=\frac{4}{3}-\frac{\bar{\alpha }}{6\pi }(2-\frac{\bar{\alpha }}{\pi })$$

The maximum equivalent stress can be approximately calculated as ${\bar{\sigma }}_{\max}=\sqrt{3}\tau_{\max}$. When it reaches material strength ${\bar{\sigma }}_{m}$, damage and a crack initiate at the bilateral notch tips B and E. Substituting Equations (2)–(5) into Equation (1), the critical load *F*_m_ acting on the off-cut segment for crack initiation is derived as follows, regarding the net acceleration as zero.

(6) $$F_{m}=\frac{\sqrt{3}\pi (D-2h{)}^{2}}{8(1+\sqrt{h/r})}\left[\frac{4}{3}-\frac{\bar{\alpha }}{6\pi }(1+\frac{\bar{\alpha }}{\pi })\right]{\bar{\sigma }}_{m}$$

*F*_m_ can be reduced by increasing the notch depth *h*, or decreasing the notch tip radius *r* and opening angle *α.* The material strength ${\bar{\sigma }}_{m}$ is dependent on the equivalent stain rate $\dot{\bar{\varepsilon }}$ and temperature *T*. In the splitting process, the equivalent strain rate $\dot{\bar{\varepsilon }}$ is defined as [24]

(7) $$\left\{ \begin{array}{l} \dot{\bar{\varepsilon }}=\frac{d(\bar{\varepsilon })}{dt}=\frac{d(\gamma /\sqrt{3})}{dt}\\ \gamma =\frac{dz_{}}{2h\tan(\alpha /2)} \end{array}\right.$$

where *γ* is the shear strain and *dz* is the displacement of the off-cut segment relative to the remainder of the bar along the *z*-direction. Therefore:

(8) $$\dot{\bar{\varepsilon }}=\frac{1}{2\sqrt{3}h\tan(\alpha /2)}\cdot \frac{dz_{}}{dt}=\frac{v_{1}^{}+v_{2}}{2\sqrt{3}h\tan(\alpha /2)}$$

The temperature rise at the fracture plane due to adiabatic shear is expressed as follows [25]:

(9) $$\Delta T=\eta \frac{\tau_{m}\gamma_{m}}{\rho C_{p}}=\eta \frac{{\bar{\sigma }}_{m}{\bar{\varepsilon }}_{m}}{\rho C_{p}}$$

where ${\bar{\varepsilon }}_{m}$ is the equivalent strain at damage initiation, *ρ* is the bar material density, and *C*_p_ is the specific heat; *η* is the fraction of plastic work dissipated to heat generation, and is set as 0.9.

The total fracture energy *W*_t_ is approximately calculated based on Equation (6)

(10) $$W_{t}=\int_{0}^{S_{t}}Fds=\frac{F_{m}S_{t}}{2}=\frac{F_{m}(D-2h){\bar{\varepsilon }}_{f}}{2}=\frac{\sqrt{3}\pi h{\left(D-2h\right)}^{3}}{16(1+\sqrt{h/\rho })}\left[\frac{4}{3}-\frac{\bar{\alpha }}{6\pi }(1+\frac{\bar{\alpha }}{\pi })\right]{\bar{\sigma }}_{m}{\bar{\varepsilon }}_{f}$$

where *S*_t_ is the splitting displacement and ${\bar{\varepsilon }}_{f}$ is the equivalent strain at complete fracture.

## 3. Experimental Tests and FE Modeling

### 3.1. Experimental Tests and Materials

**Figure 3:**
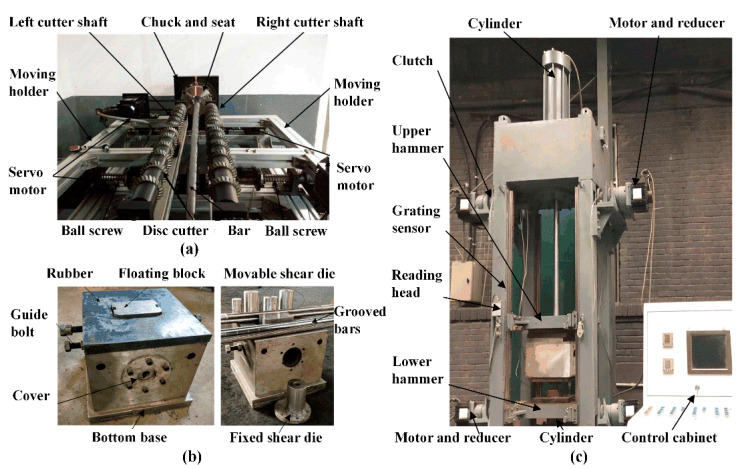
The experimental setup for notch-induced, high-speed splitting. (**a**) Symmetrically notching machine. (**b**) Double-splitting die with radial constraint. (**c**) High-speed electric-pneumatic counter hammer system.

**Figure 4:**
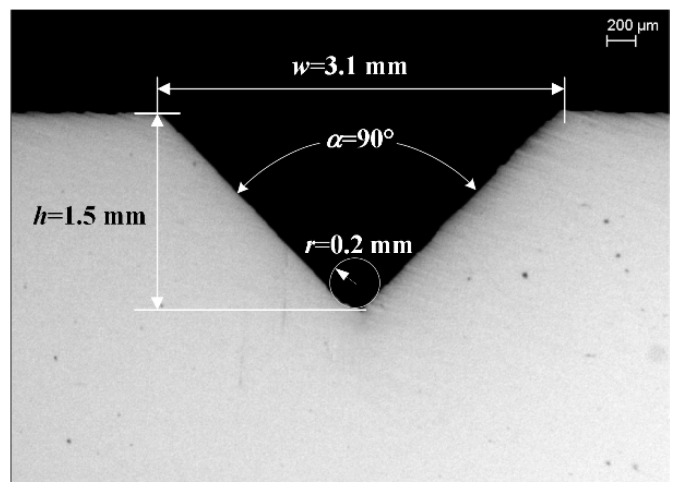
The profile of the V-shape notch.

The experimental setup is shown in Figure 3. It consists of a servo-driven, symmetrical notching machine (Figure 3a), a double-splitting die (Figure 3b), and a 16 kJ high-speed electric-pneumatic counter hammer (Figure 3c). Each hammer is pulled with steel belts driven by four 5 kW permanent-magnet synchronous motors, and locked at initial positions by pneumatic clutches so that the compressed gas is stored with great potential energy on both sides. The mass m_1_ of the upper hammer is 120 kg, the working stroke is 830 mm, and the maximum impact speed downward is 16.7 m/s at an initial gas pressure of 0.8 MPa. The total mass m_2_ of the lower hammer and the splitting die is 480 kg, the working stroke is 220 mm, and the maximum impact speed upward is 4.5 m/s. The total mass m_3_ of the floating block and the movable shear die is 28 kg. The motion characteristics of the two hammers are recorded by a pair of grating sensors. All the bars are 1000 mm long with diameter *D* of 26 mm. As shown in Figure 4, the notch depth *h* is 1.5 mm, with width w of 3.1mm, opening angle α of 90°, and tip radius *r* of 0.2 mm; the splitting length *L* is 100 mm. The axial clearance *C*_1_ and chamfer *C*_0_ are both 0.5 mm. The impact speeds of the upper and lower hammers are *v*_1_ of 9.6 m/s and *v*_2_ of 2.4 m/s, respectively, and the initial impact energy is calculated as 6912 J. Comparative tests were conducted for notched and unnotched bars of four types of materials. Each test was repeated three times.

**Figure 5:**
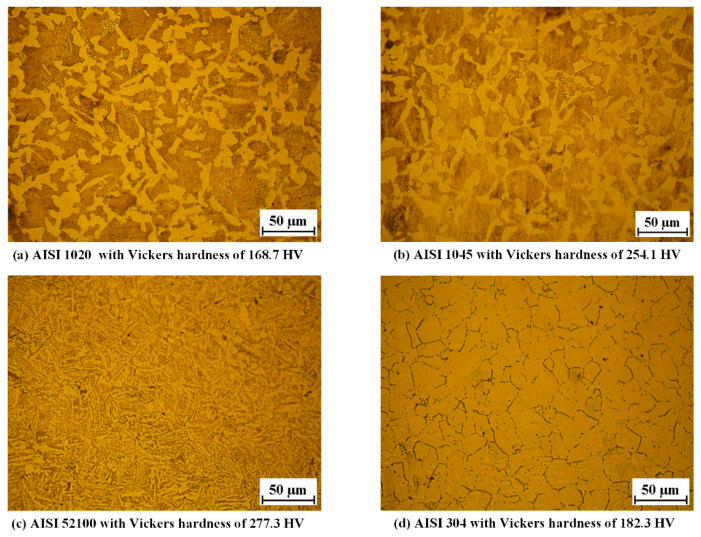
Metallographic structure and average Vickers hardness of the bar material 3.2. Details of the FE model.

The bar materials are annealed AISI 1020 steel (0.21% C), 1045 steel (0.47% C), 52100 bearing steel (1.0% C and 1.3% Cr), and 304 stainless steels (0.06% C, 18.27% Cr, 8.24% Ni, and 0.8% Mn). The metallographic structure and average Vickers harndess were obtained on the cross-section, as shown in Figure 5. The AISI 1020, 1045, and 52100 steels were processed by chemical etching with a mixed solution of nitric acid (4%) and alcohol. The metallographic structure was mixed with ferrite and pearlite. The AISI 304 was processed by electrolytic etching in the 10% oxalic acid solution with an electric current density of 1 A/cm^2^ and a duration of 90 seconds. The metallographic structure was mainly austenite. The average Vickers hardness of the cross-section was obtained using a Vickers hardness measurement apparatus with a load of 0.5 kgf and a duration of 15 s.

### 3.2. Details of the FE Model

**Figure 6:**
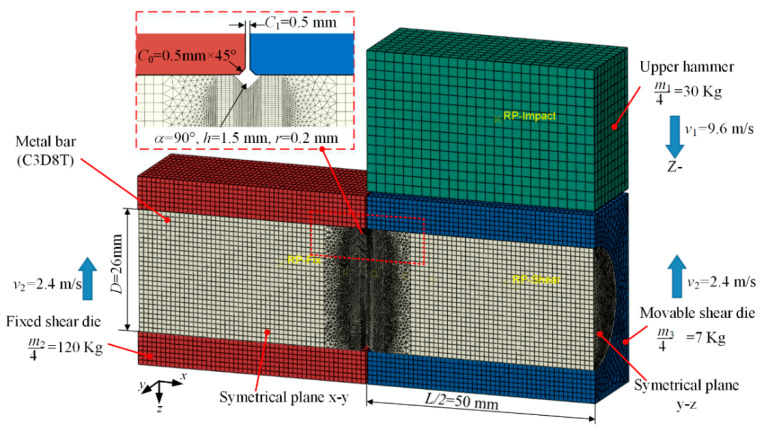
The FE model of the notch-induced, high-speed splitting of metal bar under counter impact.

A 1/4 FE model is illustrated in Figure 6, which was created using the Abaqus software coupled with a thermo-mechanical analysis. The total splitting die is integrated with the fixed shear die with mass *m*_2_ of 480 kg; the floating block is integrated with the movable shear die with mass m_3_ of 28 kg. The upper hammer, movable shear die, and the fixed shear die are modeled with the 4-node quadrilateral rigid element (R3D4 element type). The bar is modeled with 8-node coupled displacement-temperature solid elements (C3D8T element type) at the notched zones and end zones, together with 4-node coupled displacement-temperature tetrahedron element (C3D4T element type) at the transition zones. Contact properties are defined as hard contact for normal behavior and penalty friction formulation for tangential behavior; the friction coefficient is set at 0.3 for steels. The radial clearance is 0.2 mm considering the actual condition.

The plastic flow stress of the bar materials is described by the Johnson-Cook plastic model.

(11) $$\bar{\sigma }=(A+B{\bar{\varepsilon }}_{}^{n})\left[1+C\ln\left(\frac{{\dot{\bar{\varepsilon }}}_{}}{{\dot{\varepsilon }}_{0}}\right)\right]\left[1-{\left(\frac{T-T_{r}}{T_{m}-T_{r}}\right)}^{m}\right]$$

where the equivalent stress $\bar{\sigma }$ is calculated based on the equivalent plastic strain $\bar{\varepsilon }$, at specific strain rate $\dot{\bar{\varepsilon }}$ and temperature *T*. Appropriate material parameters *A*, *B*, *C*, *n,* and *m* are listed in Table 1. As the cropping of long metal bars is the first process before forging, the bar material has not yet undergone hardening and tempering, and thus, the strength and hardness are not so high [19]. ${\dot{\varepsilon }}_{0}$ is the reference strain rate at the quasi-static condition, and *T_m_* is the reference temperature, i.e., 20 °C.

Damage initiation at the notch zones is determined with the form of cumulative damage law [26], which is expressed as follows. The damage evolution and fracture process are described with the linear type displacement-based law for stiffness degradation and element deletion [27]

(12) $$\left\{ \begin{array}{l} \sum \frac{\Delta \bar{\varepsilon }}{\Delta {\bar{\varepsilon }}_{f}}=1\\ {\bar{\varepsilon }}_{f}=\left[d_{1}+d_{2}\exp(d_{3}\frac{p}{q})\right]\left[1+d_{4}\ln\left(\frac{{\dot{\bar{\varepsilon }}}_{}}{{\dot{\varepsilon }}_{0}}\right)\right]\left[1+d_{5}{\left(\frac{T-T_{r}}{T_{m}-T_{r}}\right)}^{}\right] \end{array}\right.$$

where $\Delta \bar{\varepsilon }$ is the equivalent plastic strain increment, and the equivalent failure strain ${\bar{\varepsilon }}_{f}$ is calculated based on the specific stress triaxiality p/q, equivalent strain rate $\dot{\bar{\varepsilon }}$, and temperature *T.* The material parameters *d*_1_ to *d*_5_ are listed in Table 1. 

The stress triaxiality p/q is determined as follows:

(13) $$\left\{ \begin{array}{l} p=\frac{\left(\sigma_{1}+\sigma_{2}+\sigma_{3}\right)}{3}\\ q=\sqrt{\frac{1}{2}\left[{\left(\sigma_{1}-\sigma_{2}\right)}^{2}+{\left(\sigma_{2}-\sigma_{3}\right)}^{2}+{\left(\sigma_{1}-\sigma_{3}\right)}^{2}\right]} \end{array}\right.$$

## 4. Results and Discussion

### 4.1. Fracture Behavior and Section Quality

**Figure 7:**
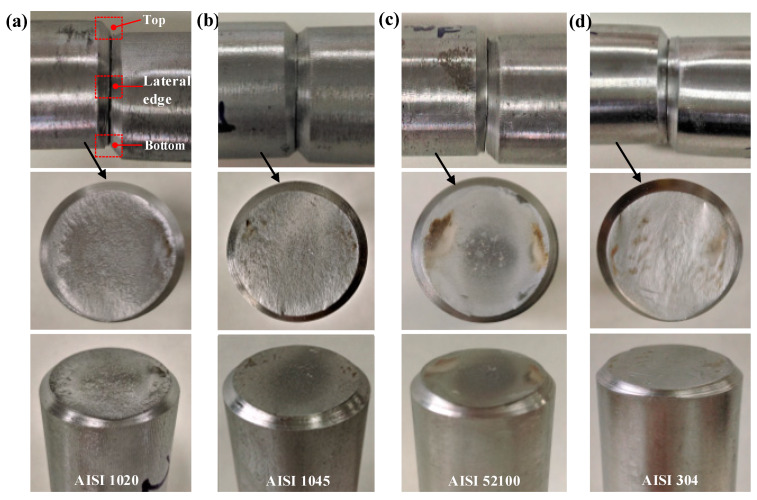
Macrofractography of bars (h = 1.5mm). (**a**) AISI 1020. (**b**) AISI 1045. (**c**) AISI 52100. (**d**) AISI 304.

**Figure 8:**
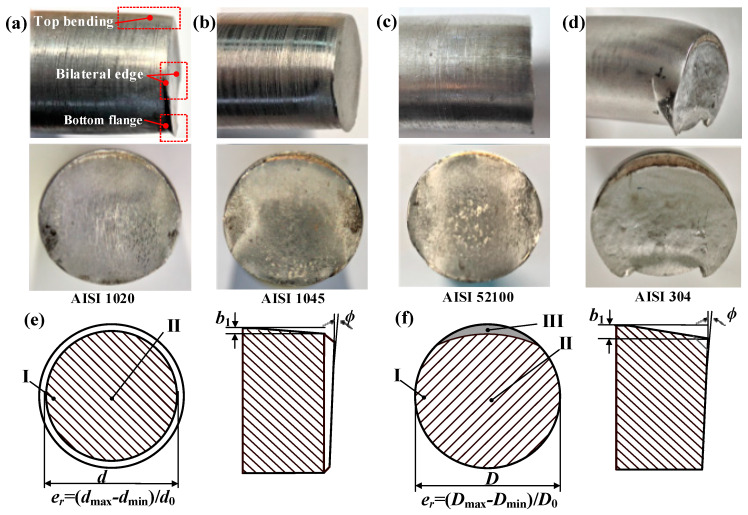
Macrofractography of bars (h = 0 mm) and quality evaluation indexes. (**a**) AISI 1020. (**b**) AISI 1045. (**c**) AISI 52100. (**d**) AISI 304. (**e**) Indexes for notched bars. (**f**) Indexes for unnotched bars.

**Figure 9:**
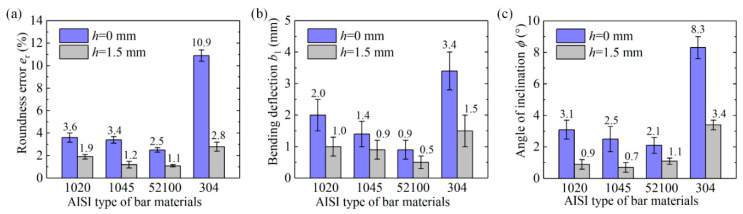
Section quality results for notched and unnotched bars. (**a**) Roundness error. (**b**) Bending deflection. (**c**) Angle of inclination.

The macrofractography is shown in Figure 7 and Figure 8, and the section quality is evaluated by indexes of roundness error *e*_r_, maximum bending deflection *b*_1_, and angle of inclination *ϕ* with standard error (Figure 9). 

Highly flat and round fracture sections are obtained for notched bars with a circumferential chamfer (Figure 7), and the material adjacent to the notch tip does not suffer much plastic distortion due to the stress concentration effect. Inward-curved crosss-sections are obtained for the unnotched bars (Figure 8), and flanges are produced at the bottom edges. Crescent-shaped shear lips [32] occur at the top edge (zone III), which reveals the material there is not only squeezed downward under radial compression stress *σ*_z_, but also undergoes large axial compression *σ*_x_ due to the bending effect. 

In a comparison of notched to unnotched bars, higher precision section quality is obtained; the roundness error *e*_r_ improves from 2.5–10.9% to 1.1–2.8%, the maximum bending deflection *b*_1_ reduces from 0.9–3.4 mm to 0.5–1.5mm, and the angle of inclination *ϕ* drops from 2.1°–8.3° to 0.7°–3.4° for the four types of material. The section quality of the notched AISI 52100 steel is prominent with roundness error *e*_r_ of 1.1%, maximum bending deflection *b*_1_ of 0.5 mm, and angle of inclination of 1.1°; low carbon steels such as AISI 304 and 1020 require a greater accumulation of three-dimensional plastic deformation for damage and cracks to occur. This new method not only effectively inhibits the plastic distortion and improves section quality, but also produces chamfered blanks, making it more suitable for the subsequent forging process to prevent the sharp external edges from damaging the forging molds.

### 4.2. FE Simulation Results

**Figure 10:**
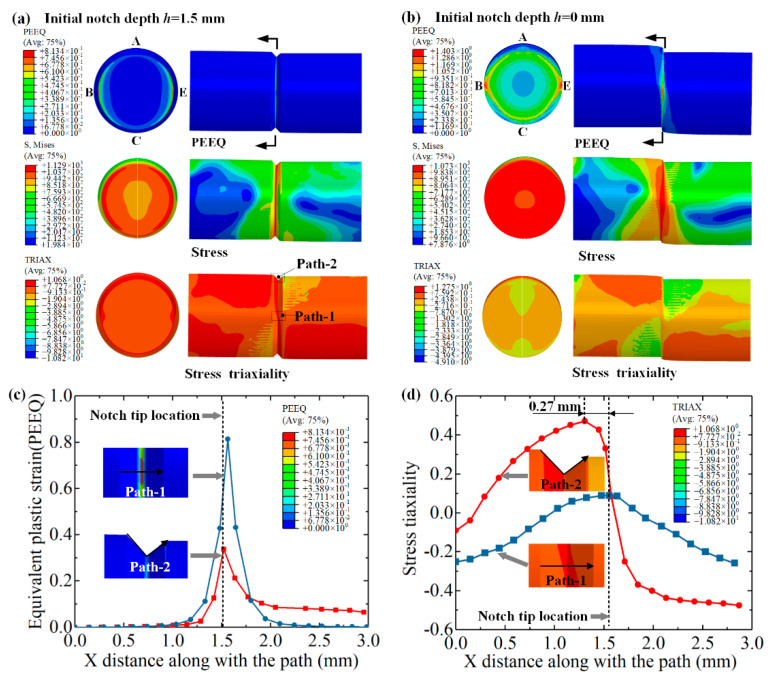
Damage initiation of notched and unnotched AISI 1045 bars. (**a**) At splitting displacement of 1.3 mm for notched bar. (**b**) At splitting displacement of 2.0 mm for unnotched bar. (**c**) Equivalent plastic strain to X distance along with path 1 and path 2. (**d**) Stree triaxiality to X distance along with paths 1 and 2.

The Mises equivalent stress, equivalent plastic strain (PEEQ), and stress triaxiality were further analyzed. Taking the notched AISI 1045 bar as an example (Figure 10a), it is obvious that stress concentrates at the notch tip, with a maximum value of 1290 MPa; damage initiates at the lateral notch tips B and E at a splitting displacement of −0.78 mm, while the rest region is under a low level of equivalent stress and plastic strain. As for the unnotched bar (Figure 10b), the stress concentrates throughout almost the whole splitting plane with a maximum value of 1073 MPa; damage initiates at the lateral notch tip B and E at a splitting displacement of 1.47 mm, which requires a more extensive accumulation of plastic deformation.

Along with path 1 at the neutral notch surface, equivalent plastic strain varies as a single-peak curve with a maximum value of 0.81 at the notch tip, and drops rapidly to 0 at the adjacent region (Figure 10c). The corresponding stress triaxiality varies as a quasi-sine curve (Figure 10d), from −0.26 to 0.08, and back to −0.26, which reveals that the material is mainly subjected to shearing at the notch tip, and under compression elsewhere. 

Along with path 2 at the top-notch surface, the peak equivalent plastic strain occurs at the notch tip with a lower value of 0.33 (Figure 10c). The stress triaxiality reaches 0.57 at the position next to the notch tip, and decrease to −0.57 at the right-side (Figure 10d). This reveals that the material is mainly subjected to shearing at the top-notch tip, with left-side under tension and right-side under compression. The FE simulation results are very consistent with the fracture behavior in the experiments.

**Figure 11:**
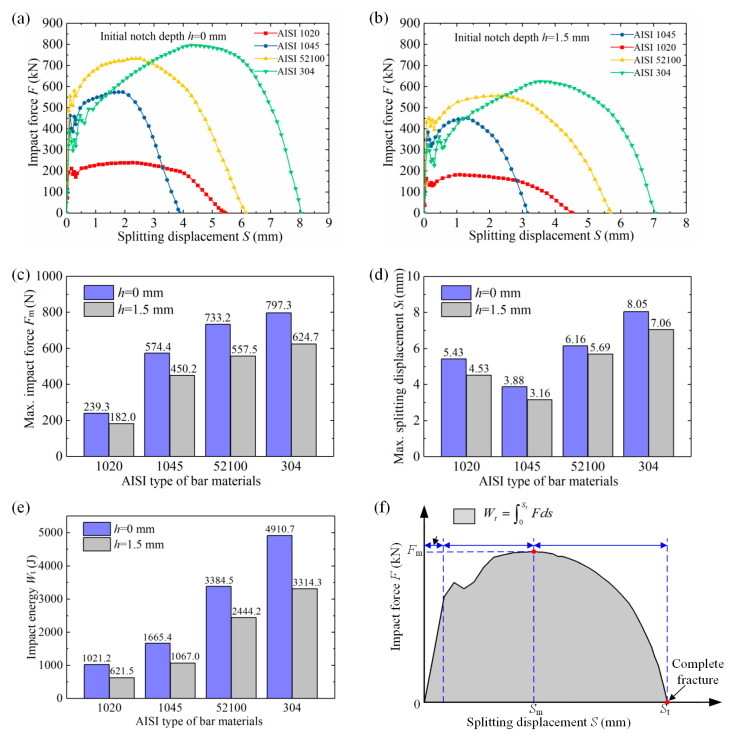
Impact force-displacement curves. (**a**) *F*-*S* curves for *h* of 0 mm. (**b**) *F*-*S* curves for *h* = 1.5 mm. (**c**) maximum impact force *F*_m_. (**d**) maximum splitting displacement *S*_t_. (**e**) Impact energy *W*_t_. (**f**) Illustration of *F*_m_, *S*_m_*, S*_t_, and *W*_t_.

Figure 11 compares the impact force-displacement curves for double-splitting, and the impact energy *W*_t_ is calculated by integrating the curves. Impact loading, as a rule, involves the oscillatory process of crack initiation. These curves were already smoothed with the method of adjacent-averaging. By introducing a surface notch, maximum impact force *F*_m_ is effectively reduced by 21.6–23.9%, the maximum displacement *S*_t_ by 7.6–8.6%, and the impact energy *W*_t_ by 27.8–39.1% for the four types of materials. Taking the AISI 1045 bar for an example, the maximum impact force *F*_m_ decreases by 17.8%, from 574.4 kN to 450.2 kN (Figure 11c); the complete fracture displacement *S*_t_ reduces by 18.6%, from 3.88 mm to 3.16 mm (Figure 11d). Impact energy *W*_t_ drops by 35.9%, from 1665.4 J to 1067.0 J (Figure 11e). The AISI 304 stainless steel exhibits a combination of high strength, good ductility, and high fracture-resistance with an impact energy *W*_t_ of 3314.3 J for notched bars; this is attributed to the content of alloying elements such as chromium (Cr), nickel (Ni), and manganese (Mn).

### 4.3. Microfracture Mechanism Analysis

**Figure 12:**
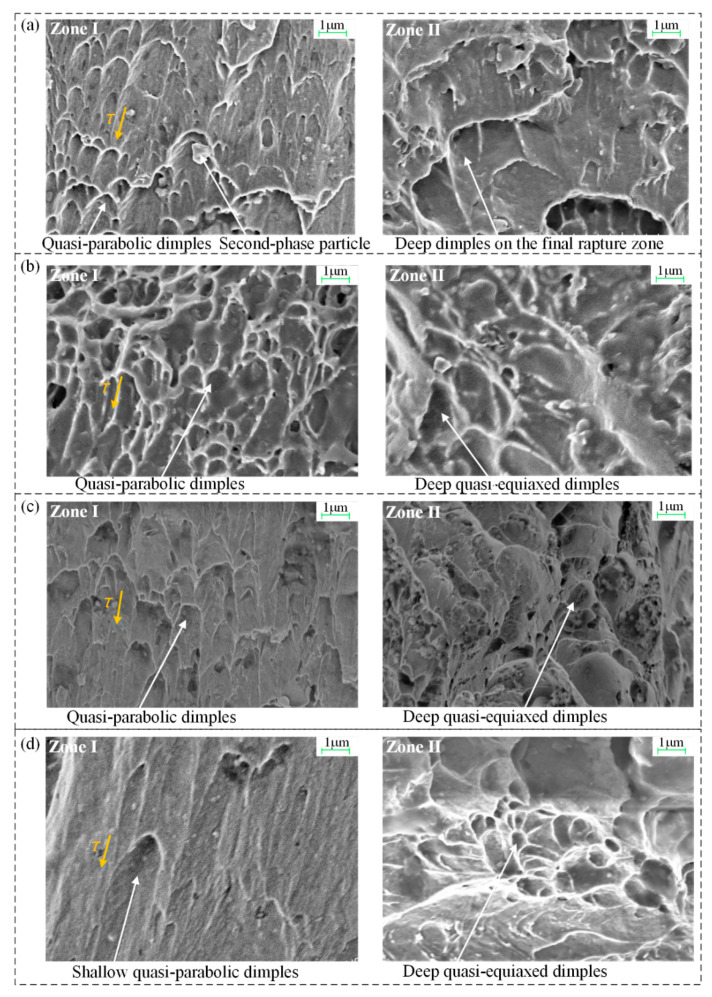
SEM fractography at surface zones I and II of different bar materials with a notch depth of 1.5mm. (**a**) AISI 1020. (**b**) AISI 1045. (**c**) AISI 52100. (**d**) AISI 304.

The microfractography of the crack initiation zone I and final rupture zone II of the surface regions were made using a Zeiss Gemini SEM 500 with a magnification factor of 10 k, as shown in Figure 12.

#### 4.3.1. Crack Initiation Zone I

The crack initiation zone I of the notched bars is characterized by quasi-parabolic shallow dimples, and the predominant fracture mechanism involves the microvoid coalescence by shear stress along with slip bands. Dispersed second-phase particles of carbide and sulfide are observed at the bottom of these dimples; slip bands are evident on the dimple wall surface. The microvoids nucleate at regions associated with second-phase particles due to localized shear strain discontinuity due to overload. Larger and more severely elongated dimples are produced in soft steels such as AISI 304 and 1020; this reveals that the pinning effect of second-phase particles is lower at the grain boundaries in these types of steel compared to AISI 52100 and 1045 steels, which contain more carbon; the smaller and closer the second-phase particles, the larger the pinning effect and the better the blocking of the dislocation movement, leading to smaller dimples.

#### 4.3.2. Final Rupture Zone II

Compared to the severely elongated dimples at zone I, the predominant topography of final rupture zone II is relatively rough and irregular, mixed with less elongated and quasi-equiaxed deeper dimples. As the dimple shape is governed by the state of stress within the material, the profile reveals that dimples are caused by the combination of tangential shear and normal stresses; the latter plays a main role in the quasi-equiaxed deeper dimples. Furthermore, deeper and larger dimples are produced in AISI 304 and 1020 steels, which is consistent with their lower strength and higher plasticity compared to AISI 52100 and 1045 steels.

## 5. Conclusions

This study presents a new notch-induced precise splitting method for high-quality cropping of metal bars on a new type of high-speed electric-pneumatic counter hammer. Currently, this method requires a different tool set for each bar diameter and blank length, and would be particularly suitable for large production runs of commonly used steel bars in the mechanical and automobile industry. Single-side cropping is also feasible to crop shorter blanks. The main conclusions are as follows:

(1) The cracks start from the notch tip and propagates through the plane induced by the notch; the remaining material is under a low level of equivalent stress and equivalent plastic strain. Along the notch surface at the neutral plane, the equivalent plastic strain varies as a single-peak curve at the notch tip, while the corresponding stress triaxiality varies as a quasi-sine curve, which reveals that the material is mainly subjected to pure shearing at the notch tip, and under compression, at other reigions.

(2) High precision fracture planes with chamfer are obtained for notched bars. The roundness error *e*_r_ improves from 2.5–10.9% to 1.1–2.8%, bending deflection *b*_1_ reduces from 0.9–3.4 mm to 0.5–1.5 mm, and angle of inclination *ϕ* drops from 2.1°–8.3° to 0.7°–3.4°. Additionally, the surface notch effectively inhibits the plastic deformation stage and reduces maximum impact force *F*_m_ by 21.6%–23.9%, maximum displacement *S*_t_ by 7.6%–18.6%, and impact energy *W*_t_ by 27.8–39.1%. 

(3) Microfractography reveals that the crack initiation zone is characterized by quasi-parabolic shallow dimples, and the predominant fracture mechanism involves microvoid coalescence by shear stress along with the slip bands. The final rupture zone II is mixed with less elongated and quasi-equiaxed deeper dimples, caused by the combination of tangential shear stress and normal tensile stress; deeper and larger dimples are produced in the AISI 304 and 1020 steels.

## Figures and Tables

**Table 1:** The Johnson-Cook plasticity and damage parameters of the bar materials.

Materials	Johnson-Cook Plasticity Parameters						Damage Parameters				
	*A* (Mpa)	*B* (MPa)	*n*	*m*	*C*	${\dot{\varepsilon }}_{0}$	*d*_1_	*d*_2_	*d*_3_	*d*_4_	*d*_5_
1020 [28]	213	53	0.345	0.81	0.055	0.004	0.05	3.44	−2.12	0.002	0.61
1045 [29]	506	320	0.28	1.06	0.064	1	0.1	0.76	−1.57	0.005	−0.84
52100 [30]	774.78	134	0.37	3.171	0.018	1	0.0368	2.34	−1.484	0.0035	0.411
304 [31]	310	1000	0.65	1	0.07	0.1	0.53	0.5	−6.8	−0.014	0.0
